# The *KIT* Gene Is Associated with the *English Spotting* Coat Color Locus and Congenital Megacolon in Checkered Giant Rabbits (*Oryctolagus cuniculus*)

**DOI:** 10.1371/journal.pone.0093750

**Published:** 2014-04-15

**Authors:** Luca Fontanesi, Manuela Vargiolu, Emilio Scotti, Rocco Latorre, Maria Simonetta Faussone Pellegrini, Maurizio Mazzoni, Martina Asti, Roberto Chiocchetti, Giovanni Romeo, Paolo Clavenzani, Roberto De Giorgio

**Affiliations:** 1 Department of Agricultural and Food Sciences, Division of Animal Sciences, Laboratory of Livestock Genomics, University of Bologna, Bologna, Italy; 2 Centre for Genome Biology, University of Bologna, Bologna, Italy; 3 Health Sciences and Technologies-Interdepartmental Center for Industrial Research, University of Bologna, Bologna, Italy; 4 Department of Veterinary Medical Science, University of Bologna, Bologna, Italy; 5 Department of Medical and Surgical Sciences, Centro Unificato di Ricerca Biomedica Applicata (C.R.B.A.), St. Orsola-Malpighi Hospital, University of Bologna, Bologna, Italy; 6 Department of Experimental and Clinical Medicine, Section of Anatomy and Histology, University of Florence, Florence, Italy; 7 Department of Medical and Surgical Sciences, Medical Genetics Unit, St. Orsola-Malpighi Hospital, University of Bologna, Bologna, Italy; Institut Jacques Monod, France

## Abstract

The *English spotting* coat color locus in rabbits, also known as *Dominant white spotting* locus, is determined by an incompletely dominant allele (*En*). Rabbits homozygous for the recessive wild-type allele (*en*/*en*) are self-colored, heterozygous *En/en* rabbits are normally spotted, and homozygous *En/En* animals are almost completely white. Compared to vital *en*/*en* and *En/en* rabbits, *En/En* animals are subvital because of a dilated (“mega”) cecum and ascending colon. In this study, we investigated the role of the *KIT* gene as a candidate for the *English spotting* locus in Checkered Giant rabbits and characterized the abnormalities affecting enteric neurons and c-kit positive interstitial cells of Cajal (ICC) in the megacolon of *En/En* rabbits. Twenty-one litters were obtained by crossing three Checkered Giant bucks (*En/en*) with nine Checkered Giant (*En/en*) and two *en/en* does, producing a total of 138 F1 and backcrossed rabbits. Resequencing all coding exons and portions of non-coding regions of the *KIT* gene in 28 rabbits of different breeds identified 98 polymorphisms. A single nucleotide polymorphism genotyped in all F1 families showed complete cosegregation with the *English spotting* coat color phenotype (*θ* = 0.00 LOD  = 75.56). *KIT* gene expression in cecum and colon specimens of *En/En* (pathological) rabbits was 5–10% of that of *en/en* (control) rabbits. *En/En* rabbits showed reduced and altered c-kit immunolabelled ICC compared to *en/en* controls. Morphometric data on whole mounts of the ascending colon showed a significant decrease of HuC/D (*P*<0.05) and substance P (*P*<0.01) immunoreactive neurons in *En/En* vs. *en/en*. Electron microscopy analysis showed neuronal and ICC abnormalities in *En/En* tissues. The *En/En* rabbit model shows neuro-ICC changes reminiscent of the human non-aganglionic megacolon. This rabbit model may provide a better understanding of the molecular abnormalities underlying conditions associated with non-aganglionic megacolon.

## Introduction

Early genetics studies, started soon after the rediscovery of Mendel's laws, have identified several loci affecting coat color in the European rabbit (*Oryctolagus cuniculus*) (reviewed in [1]–[3]). More recently, molecular genetics studies in this species have established that mutations in the tyrosinase (*TYR*), melanocortin 1 receptor (*MC1R*), agouti signalling protein (*ASIP*), melanophilin (*MLPH*) and tyrosinase-related protein 1 (*TYRP1*) genes are the causative events of alleles described at the *albino*, *extension*, *agouti*, *dilute* and *brown* coat color loci, respectively [4]–[9]. Despite recent advances, additional coat color loci remain to be characterized at the molecular level in rabbits. One of them is the *English spotting* locus, also known as *Dominant white spotting* locus, that classical crossbreeding experiments showed to be determined by an incompletely dominant allele (*En*). Rabbits homozygous for the recessive non-mutated allele (*en*/*en*) are self-colored (not spotted). Heterozygous *En/en* rabbits are normally spotted and possess far larger patches of colored fur compared to the homozygous *En/En* animals that have a reduced spotted pattern [2], [10]. The *En/en* genotype is selected for show purposes and a few breeds/strains, like the Checkered Giant and English spot, have recognized standards that are considered to result from this allele combination, even if their spotted patterns are different. *En/En* rabbits are subvital compared to vital heterozygous *En/en* rabbits because dominant homozygous animals are affected by an underlying megacolon [2], [11], [12]. This defect is recessive since it is not observed in *En/en* rabbits, and with putative incomplete penetrance, probably modified by environmental conditions (e.g. diet, stressors and ageing) or other modifier genes. The few studies that have investigated the etiopathogenesis of this form of megacolon suggest the involvement of enteric nervous system (ENS) abnormalities throughout the colon [13], but its characterization is still incomplete [11], [12].

Apart from genetic knock-out mouse models [14], [15], few naturally occurring animal models are available for megacolon. Phenotypes cover the wide spectrum of gut neuromuscular derangements underlying megacolon and are not limited to enteric aganglionosis. Although particularly useful for physiological studies, the rabbit has not often been used as a model to study the molecular mechanisms of human gastrointestinal pathologies.

Genes that affect coat color in other mammals have been implicated in the pathogenesis of aganglionic megacolon, and the *English spotting* locus may account for a similar condition in the rabbit. Previous evidence indicates that inactivation of the endothelin receptor B (*EDNRB*) gene in the mouse causes aganglionic megacolon with spotted coat coloring [16], [17], and *EDNRB* mutations are known to be associated with Hirschsprung disease in patients with Shah-Waardenburg syndrome [18], [19]. However, previous data from our group excluded *EDNRB* as the causative gene in the *English spotting* locus in Checkered Giant rabbits [20], prompting our attention to other genes involved in melanogenesis and in the regulation of cell proliferation, differentiation, migration and survival.

One of these genes is the v-kit Hardy-Zuckerman 4 feline sarcoma viral oncogene homolog (*KIT*) gene that encodes the mast/stem cell growth factor receptor. This is a large protein with an extracellular domain consisting of 5 Ig-like sub-domains, a transmembrane region, and a tyrosine kinase domain [21]. *KIT* expression in the gut musculature is prominent only in interstitial cells of Cajal (ICC). ICC are known to play important roles in gut motility and their abnormalities are associated with several gastrointestinal motility disorders [22], [23]. KIT is also involved in driving the migration of melanocytes from the neural crest along the dorsolateral pathway to colonize the final destination in the skin [24]. A large number of mutations in the *KIT* gene affecting pigmentation have been already described in humans, mice, pigs, cattle, horses, cats and dogs [25]–[37].

In this study we investigated the role of the *KIT* gene as a candidate for the *English spotting* locus in Checkered Giant rabbits. Our results demonstrate that this gene is associated with the coat color locus and may play an important role in determining the associated megacolon defect in *En/En* rabbits by altering ICC and the enteric neuronal component.

## Methods

### Ethics statement

The experiments reported in this study were approved by the Ethics Committee for Experiments on Animals of the University of Bologna, protocol no. 50932-X/10, and comply with Italian and European Union regulations. All efforts were made to minimize the number of animals used in this study and their suffering.

### Animals

**Figure 1:**
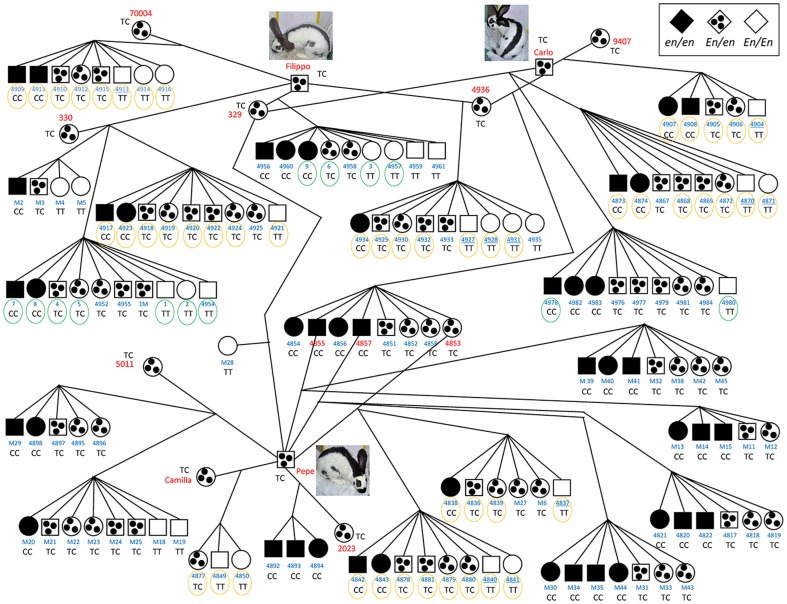
F1 and backcrossed families obtained from Checkered Giant parental animals. Black squares and circles  =  rabbits with *en/en* genotype at the *English spotting* locus; spotted squares and circles  =  rabbits with *En/en* genotype; white squares and circles  =  rabbits with *En/En* genotype. ID of the animals is reported in blue or red (for those used as parental animals). The genotype of the g.93948587T>C SNP is reported for all animals. Rabbits that are circled in green have been used to collect specimens for anatomo-histochemical, gene expression analyses and resequencing (rabbits with n. 1–9). Rabbits that are circled in orange have been inspected after slaughtering. Animals with underlined ID showed hard feces in the intestine.

**Figure 2:**
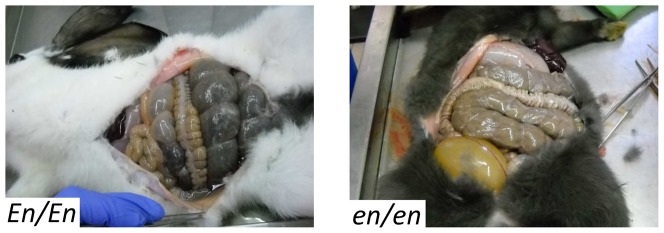
Typical appearance of the intestine in *En/En* and *en/en* weaned rabbits. Macroscopic features of the gut in the rabbit model investigated in this study. Note the markedly dilated ascending colon in *En/En* (pathological) compared to *en/en* rabbits (control).

Twenty-one litters were obtained by crossing three Checkered Giant bucks (with *En/en* genotype) with i) nine Checkered Giant does (with *En/en* genotype), producing a total of 113 rabbits, and ii) two *en/en* does, producing a total of 25 other rabbits (Figure 1). Animals were housed in a controlled environment and had free access to food and water throughout the study. Photographs were taken of all animals just before weaning 35–40 days after birth. Thirteen 35–40 days old F1 rabbits (4 of the *en/en* genotype, 2 females and 2 males; 3 of the *En/en* genotype, 2 females and 1 male; and 6 of the *En/En* genotype, 3 females and 3 males; all from 3 families; Figure 1) were euthanized to collect specimens for sub-sequent anatomo-histochemical and gene expression analyses (see below). Animals were euthanized by an intracardiac injection of Tanax (0.5 ml kg BW; Intervet, Milan, Italy) following deep anaesthesia with an intravenous injection of 0.4 ml/kg of acepromazine maleate (Prequillan, FATRO, Bologna, Italy) and 1.5 ml/kg body weight (BW) of xylazine (Rompun, Bayer, Leverkusen, Germany). The typical appearance of the intestines in young *En/En* and *en/en* rabbits is shown in Figure 2. Other rabbits (Figure 1) were slaughtered in a commercial slaughterhouse at age 70–75 days and the intestine was visually inspected in 50 animals (15 *En/En*, 23 *En/en*, 12 *en/en*) for evident signs of megacolon. Photographs of these animals were taken before slaughtering and no substantial differences were noted before weaning and before slaughtering. Blood, liver, ascending colon, cecum, and/or lymph node samples were collected for subsequent analyses from animals after slaughter, euthanasia or natural death. Other rabbits with different *English spotting* genotypes were used for resequencing (see below). Hair roots were collected from additional rabbits of different breeds registered to the herd book of the National Rabbit Breeder Organization (ANCI) (Table 1). These animals were used for genotyping the g.93948587T>C SNP.

**Table 1:** Breeds and rabbits genotyped for the *KIT* g.93948587T>C single nucleotide polymorphism.

Breed (No. of animals)	Coat color and [genotype at the *English spotting* locus]	*KIT* g.93948587T>C genotype^1^		
		T/T	T/C	C/C
Blue Vienna (13)	Self colored: dark blue [*en/en*]	9	2	2
Californian (20)	Albino with spots at the extremities [*en/en*]	1	3	16
Champagne d'Argent (12)	Self colored: silver as surface color and black as under-color [*en/en*]	-	-	12
Checkered Giant (20)	Checkered pattern - white with black or blue markings – [*En/en*]	-	20	-
Dutch (6)	Black with white belt [*en/en*]	1	2	3
English Spot (8)	English spot pattern - white with black or blue markings [*En/en*]	-	-	8
Giant Chinchilla (12)	Self colored: chinchilla [*en/en*]	-	3	9
Giant Grey (6)	Self colored: wild-grey [*en/en*]	3	3	-
Giant White (2)	White albino [?]	4	-	-
Japanese (2)	Self colored: Japanese (tricolor) [*en/en*]	-	-	2
New Zealand White (4)	White albino [?]	-	-	4
Rhinelander (3)	Checkered pattern - white with black and yellow markings [*En/en*]	-	3	-

1 The number of rabbits with the different genotypes is reported.

### Resequencing of the rabbit KIT gene

Genomic DNA was extracted from blood or liver using the Wizard Genomic DNA Purification kit (Promega Corporation, Madison, WI, USA) or from hair roots as already described [38]. Genomic DNA for PCR amplification and sequencing was obtained from i) 3 *En/En*, 3 *En/en* and 3 *en/en* F1 rabbits obtained from 2 different families (rabbits numbered 1–9 in Figure 1), ii) from 5 unrelated Checkered Giant rabbits (*En/en*), iii) from 14 rabbits of different breeds (2 Californian, 1 Champagne d'Argent, 3 Giant Chinchilla, 2 Giant Grey, 2 Japanese and 4 Vienna Blue) with *en/en* genotype. PCR primers were designed for the rabbit *KIT* gene (Ensembl number ENSOCUG00000007086, reported in the oryCun2.0 genome version of the *Oryctolagus cuniculus*, assembled by the Broad Institute in the context of the Mammalian Genome Project [39]) to amplify all reported exons (except part of the uncompleted assembled exon 12 that was subsequently resequenced after reverse transcription-PCR; see below), portions of intronic regions downstream or upstream to recognized exons, and 5′- and 3′-untranslated regions (Table S1 and Figure S1). PCR was carried out using a PTC-100 (MJ Research, Watertown, MA, USA) or a 2700 thermal cycler (Applied Biosystems, Foster City, CA, USA) in a 20 µL reaction volume containing ∼50 ng genomic DNA, 1 U DNA EuroTaq DNA polymerase (EuroClone Ltd., Paington, Devon, UK) or AmpliTaq Gold DNA Polymerase (Applied Biosystems), 1X *Taq* PCR buffer, 2.5 mM dNTPs, 10 pmol of each primer and 1.5–3.0 mM of MgCl_2_ (Table S1). Thirty-five amplification cycles of 30 sec at 95°C, 30 sec at 57–60°C, PCR cycles were preceded by 5 min at 95°C (preliminary denaturation step) and followed by 5 min at 72°C (final elongation step) (Table S1). The amplified fragments were treated with 1 µL of ExoSAP-IT (USB Corporation, Cleveland, Ohio, USA) for 15 min at 37°C and then sequenced using a cycle sequencing protocol with PCR primers used for the amplification and the Big Dye v3.1 cycle sequencing kit (Applied Biosystems). Sequencing reactions were purified and then loaded on ABI3100 Avant or ABI3730 capillary sequencers (Applied Biosystems). Sequences were aligned and polymorphisms were detected using CodonCode Aligner (http://www.codoncode.com/aligner) with the genomic rabbit *KIT* sequence used as reference. All sequence chromatograms were visually inspected. The nomenclature of identified polymorphisms was based on system coordinates of the *KIT* gene sequence ENSOCUG00000007086 in the oryCun2.0 genome version (Ensembl release 74; December 2013). *In silico* functional analysis of the identified missense mutation was carried out using PANTHER [40] and SIFT [41]. Potential effects of other mutations on splicing was obtained with ESEfinder 3.0 [42] and the Berkeley Drosophila Genome Project splicing site analysis tool (http://www.fruitfly.org/seq_tools/splice.html).

### Genotyping and linkage analysis

A SNP identified in *KIT* exon 5 (g.62610715T>C) was genotyped by PCR-RFLP in all parental, F1 and backcrossed animals of the twenty-one litters (Figure 1) and in rabbits of different breeds (Table 1). The amplified products covering exon 5 (Table S1) were digested with 2 U of *Fsp*BI restriction enzyme (Thermo Scientific-Fermentas, Vilnius, Lithuania), at 37°C overnight in a 25 µL reaction volume including 5 µL of PCR product and 1X restriction enzyme buffer. The produced DNA fragments were electrophoresed on 10% 29∶1 polyacrylamide:bis acrylamide or 2% agarose gels in 1X TBE buffer and visualized with 1X GelRed Nucleic Acid Gel Stain (Biotium Inc., Hayward, CA, USA). Electrophoretic patterns for the two alleles were the following: allele T, three fragments of 302+148+47 bp; allele C, two fragments of 450+47 bp.

Linkage between coat color phenotypes and g.93948587T>C genotypes in the F1 rabbits was evaluated with the LODS program (Linkage Utility Programs, Rockefeller University).

### RNA extraction, Reverse Transcription-PCR and qPCR analysis

RNA was extracted from snap-frozen tissues of euthanized rabbits (ascending colon, cecum, liver and lymph nodes) using Trizol reagent (Life Technologies/Invitrogen, Carlsbad, CA, USA), and cDNA was prepared with GoTaq 2-Step RT-qPCR System (Promega Corporation) following manufacturers' instructions.

Reverse Transcription-PCR (RT-PCR) assays were carried out using the following primer pairs: KIT_cDNA_1, KIT_cDNA_2, KIT_cDNA_3, KIT_cDNA_4, KIT_cDNA_5 and KIT_cDNA_6 (Table S1). After an initial denaturation at 95°C for 5 min, 40 cycles were performed in a 2700 thermal cycler (Applied Biosystems) with the following program: 94°C for 1 min, 60°C for 1 min, 72°C for 2 min. The RT-PCR products were analyzed on 2% agarose gels stained with 1X GelRed Nucleic Acid Gel Stain (Biotium Inc.). All PCR products were verified by direct sequencing carried out as described above.

Real time gene expression levels of *KIT* and hypoxanthine-guanine phosphoribosyltransferase (*HPRT*) used as reference [43] were measured in the *En/En* and *en/en* euthanized rabbits on a PCR 7500 Fast Real Time System (Applied Biosystems) through relative quantification using SYBR Green chemistry and a specific amplification protocol (initial denaturation at 95°C for 10 min and 40 cycles at 95°C for 15 s and 60°C for 1 min) with primer pairs KIT_cDNA_2, KIT_cDNA_5, and KIT_cDNA_6 (Table S1). Analyses were carried out in quintuplicate for each primer pair/animal/specimen combination. All target fragments were normalized to the corresponding endogenous control (*HPRT*) using the Δ*C_t_* comparative method. Data output were expressed as averaged fold difference expression levels between *En/En* and *en/en* rabbits and compared using one-tailed unpaired *t*-test.

### Whole mount processing and cryosections

Whole mount processing was carried out as previously described [44]. Briefly, the large intestine of euthanized rabbits was gently removed and a segment (about 25 cm) of the ascending colon was collected and immediately immersed for 15 min in phosphate buffered saline (PBS) 0.01 M pH 7.2, containing the L-type calcium channel blocker nicardipine 0.1% as a muscle relaxant (10^−5^ M; Sigma-Aldrich, Milan, Italy). A portion of ∼20 cm was dissected from each ascending colon segment and opened along mesenteric border. The tissue was vigorously flushed out with PBS and pinned taughtly on balsa wood with the mucosal surface facing down. Specimens were subsequently fixed in Zamboni's fixative (2% formaldehyde, 0.2% picric acid in 0.1 M PBS) at 4°C overnight. Afterwards, they were removed from the balsa wood and washed in dimethyl sulfoxide (DMSO, Sigma-Aldrich; 3×10 min), followed by washing in PBS (3×10 min). All tissues were stored at 4°C in PBS containing sodium azide (0.1%) until they were processed to obtain whole-mount preparations. Three small portions of tissue (∼2 cm^2^) were isolated from each ascending colon segment, from cranial (1 cm), medium (7 cm) and caudal (20 cm) positions.

A portion of the ascending colon (2 cm^2^), near the samples previously collected for whole mount, was removed, pinned to balsa wood without stretching, and fixed overnight in 4% paraformaldehyde at 4°C. On the following day, tissues were transferred to a mixture of PBS–sucrose-azide (PBS containing 0.1% sodium azide and 30% sucrose as cryoprotectant) and stored at 4°C overnight. Tissues were then immersed in a mixture of PBS–sucrose-azide and optimal cutting temperature compound (OCT; Tissue Tek, Sakura Finetek Europe, Zoeterwoude, The Netherlands) at a ratio of 1∶1 for an additional 24 h before being embedded in 100% OCT and frozen in isopentane cooled in liquid nitrogen. Ten-µm thick transverse sections were cut, placed onto polarized slides and stored at −80°C until staining was performed.

### Immunohistochemistry

Cryostat sections and whole mount preparations were processed for single- and double-indirect immunofluorescence. Whole mount preparations were incubated in 10% normal donkey serum in PBS containing 1% TRITON X-100 for 1 h at room temperature (RT) to reduce non-specific binding of the secondary antibody and to permeabilize the tissue to the antisera. Whole mounts were then incubated at 4°C in a humid chamber for 48 h in a mixture of primary antibody against the human neuronal protein (HuC/D) pan-neuronal marker in association with the following primary antisera: neuronal nitric oxide synthase (nNOS), substance P (SP) and calcitonin gene-related peptide (CGRP) (Table S2). Cryostat sections were incubated in 10% of the appropriate normal serum in PBS containing 1% TRITON X-100 for 1 h at RT and, successively, incubated overnight in a humid chamber at 4°C with a mixture of c-kit and nNOS primary antibodies (Table S2). After washing (3×10 min) in PBS, whole mount preparation were incubated for 3 h at RT in a humid chamber in a mixture of fluorescein isothiocyanate (FITC)-conjugated, tetramethyl rhodamine isothiocyanate (TRITC)-conjugated, Alexa Fluor 594-coniugated and Alexa Fluor 488-coniugated secondary antibodies all diluted in PBS. Cryosections (after washing 3×10 min in PBS) were incubated for 1 h at RT in a humid chamber in a mixture of TRITC-conjugated and Alexa Fluor 488-coniugated secondary antibodies all diluted in PBS (Table S2). Finally, whole mount preparations and the cryosections were washed in PBS and coverslipped with buffered glycerol (pH 8.6).

Preparations were examined with a microscope (Axioplan epifluorescence microscope, Carl Zeiss, Oberkochen, Germany) equipped with the appropriate filter cubes to discriminate between FITC, TRITC, Alexa 488 and Alexa 594 fluorescent. For each ascending colon segment, the density of total HuC/D, nNOS, SP and CGRP neurons was counted in 12 microscope fields (each field, 0.28 mm^2^) in cranial (1 cm), medium (7 cm) and caudal (20 cm) whole mount preparations which had previously been determined by means of two orthogonal coordinates taken from a table of random numbers and measured on the movable stage of the microscope. Therefore, for each animal, a mean area of 10.08 mm^2^ was evaluated. The images were recorded by a digital photocamera (DMC, Polaroid, Cambridge, MA, USA) and a software program (DMC2, Polaroid). Adjustments to contrast and brightness were performed using software programs (Corel Photo Paint and Corel Draw, Ottawa, ON, Canada).

All mean data, subdivided for *En/En* (n = 6) and *en/en* (n = 4) rabbits, were tested for normality with a Shapiro-Wilk W test. As the Shapiro-Wilk W test demonstrated a normal distribution, comparisons between the two groups (of different number) were evaluated with parametric unpaired *t*-test. Analyses were performed using Graph Prism 4 software (GraphPad Software, Inc., La Jolla, CA, USA) statistic, with *P*<0.05 used to define statistical significance. Data are expressed as mean ± standard error of the mean (SEM).

### Electron microscopy

Tissue was collected from cecum, ascending, transverse and descending colon of both *En/En* and *en/en* animals. We used colonic strips, 1 mm X 3 mm long and containing the *Muscularis propria* plus a small portion of the *Tunica submucosa*. The strips were immediately cut after the excision and fixed for 6 h in a solution of 2% glutaraldehyde 0.1 M in cacodylate buffer (pH 7.4). After four rinses in the cacodylate-buffered solution containing 0.22 M sucrose, the strips were post-fixed for 1 h in 1% OsO_4_ in 0.1 M phosphate buffer. Dehydration was carried out in graded ethanol and the strips were then embedded in Epon using flat moulds to obtain sections with the circular muscle cut in cross-section. Semi-thin sections, obtained with a LKB-NOVA ultramicrotome (Stockholm, Sweden), were stained with a solution of toluidine blue in 0.1 M borate buffer and then observed under a light microscope to select areas away from the strip edges and with no apparent signs of mal-fixation or processing artefacts. Ultra-thin sections of these selected areas were obtained with the LKB NOVA ultramicrotome using a diamond knife and stained with a saturated solution of uranyl acetate in methanol (50∶50) per 12 min at 45°C, followed by an aqueous solution of concentrated bismuth subnitrate per 10 min at room temperature. At least 10–20 ultra-thin sections from all four strips of each animal were examined under a JEOL 1010 electron microscope (JEOL, Tokyo, Japan) and photographed.

## Results

### Coat color segregation in Checkered Giant rabbit families

**Figure 3:**
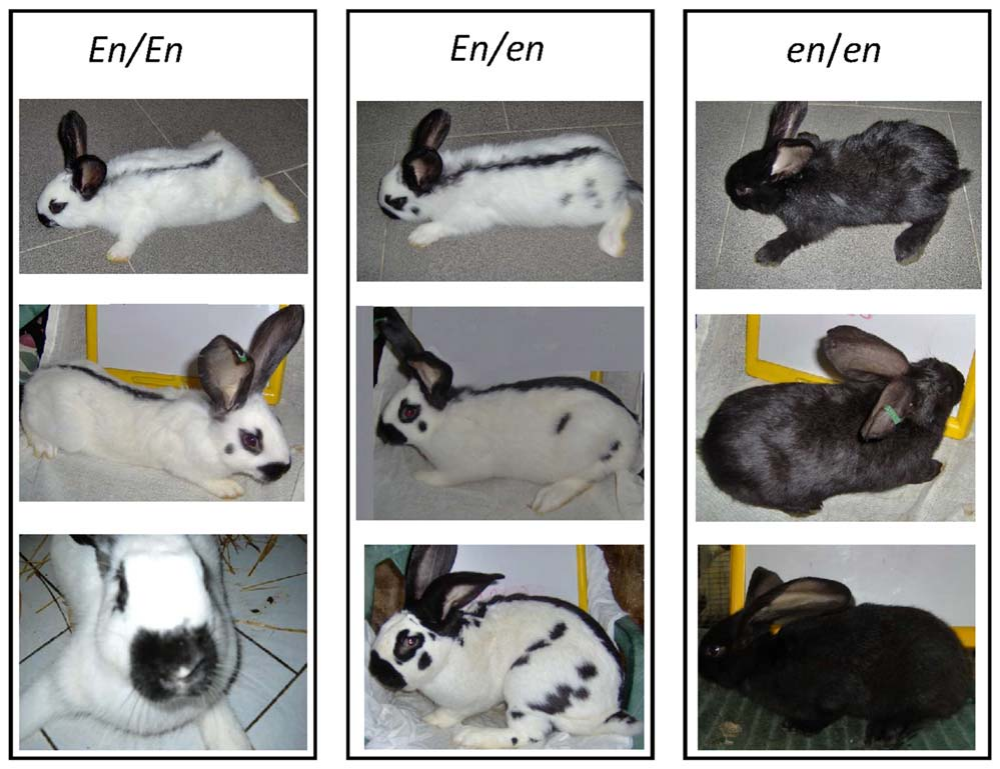
Rabbits with *En/En*, *En/en* and *en/en* genotypes at the *English spotting* locus. The genotypes are identified by the three coat color phenotypes, respectively.

Crossing three Checkered Giant bucks with nine Checkered Giant does we obtained a total of 29 *En/En*, 53 *En/en* and 31 *en/en* rabbits (Figure 1). The genotype of F1 rabbits was assigned by their coat colors, despite variability of the spotting pattern evident in *En/en* and *En/En* animals (Figure 3). These two genotypes were clearly distinguishable because *En/En* rabbits have depigmented muzzles as a typical sign, as well as a usually thinner back colored line than in *En/en* animals and absence of spots on the cheeks (or just one small spot per side) and upper legs (Figure 3). The ratio of the three genotype classes was not different from the 1∶2∶1 Mendelian ratio (ϰ^2^ = 0.326; P = 0.854), as expected from the genotype of the parental animals (*En/en*) and the partial dominance of the *En* allele [2]. Crossing an *En/en* buck with *en/en* does yielded four litters with a total of 13 *en/en* and 12 *En/en* F1 rabbits. Mendelian ratio of the two genotypes was 1∶1, as expected. No difference between the two sexes within the three genotypes was observed, confirming the autosomal location of the *English spotting* locus.

All *En/En* adult slaughtered rabbits showed evident signs of megacolon with, in most cases (10/15) hard, dry feces (Figure S2), whereas none of the *En/en* and *en/en* animals showed macroscopic evidence of this defect. Furthermore, ancillary studies demonstrated a lack of neuromuscular and ICC changes in both *En/en* as well as *en/en* rabbits (see below). Thus, we based our subsequent tests on the two main phenotypes, i.e. *En/En* (pathological) and *en/en* (control) rabbits.

### Association between the English spotting locus and a KIT gene marker

To evaluate whether the *KIT* gene could be associated with the *English spotting* locus, we first sequenced a few regions of this gene to identify useful DNA markers. Sequencing was carried out on amplified products obtained from 3 rabbits for each of the three genotypes from two half-sib F1 families. A synonymous SNP identified in exon 5 (g.93948587T>C) was selected for genotyping all rabbits of the F1 and backcrossed families, including parents (Figure 1). Complete co-segregation of this marker and coat color phenotypes was observed (*θ* = 0.00 LOD  = 75.56). All animals with the T/T genotype were *En/En* at the *English spotting* locus, all rabbits with the C/C genotype were self-colored (*en/en*), and all T/C heterozygous rabbits were *En/en*, like all Checkered Giant parents. Twenty other unrelated Checkered Giant and 3 Rhinelander (tricolor with Checkered pattern) rabbits that were genotyped at this polymorphic site had the T/C genotype (Table 1). Rabbits of other breeds with different coat color had all three genotypes suggesting that this SNP was in complete linkage disequilibrium with the *English spotting* locus in Checkered Giant rabbits and Rhinelander rabbits with Checkered patterns. In particular, all rabbits of the English spot breed had C/C genotype at the g.93948587T>C SNP (Table 1). It is worth mentioning that English spot rabbits have a different spotted pattern than Checkered Giant rabbits, with many small spots on body sides [7]. Rabbits of the Dutch breed were not fixed for any genotype (Table 1).

### Identification of other polymorphisms in the KIT gene

According to the results obtained with the g.93948587T>C SNP indicating that the *KIT* gene may be involved in determining the *English spotting* phenotype in Checkered Giant rabbits, we resequenced a total of 9154 bp of this gene (EMBL/GenBank accession numbers KJ495952 and KJ495953), including all annotated 21 exons and adjacent non-coding regions (Figure S1). Resequencing was carried out on the same 9 F1 rabbits used to identify the g.93948587T>C SNP (3 *En/en*, 3 *En/en*, and 3 *en/en*), and 19 additional rabbits of different breeds, five of which were *En/en* Checkered Giant rabbits and the rest were presumed to be *en/en*. A total of 98 polymorphisms were identified (90 SNP, 7 indels and one microsatellite), of which 49 were in introns, 32 in non-coding exonic regions and 17 in coding regions (Table S3). All SNPs in the coding regions were synonymous substitutions except one missense mutation (p.N213D) in exon 4. PANTHER and SIFT analysis indicated that this amino acid substitution is tolerated (Table S3). None of the changes detected in the mutation screening were predicted to have potential effects on splicing by *in silico* analysis using Berkeley Drosophila Genome Project and ESEfinder 3.0.

All Checkered Giant and *En/en* F1 rabbits carried two haplotypes spanning the whole *KIT* gene (Table S3). Haplotype 1 was homozygous in all three *En/En* F1 rabbits. Interestingly, this haplotype was also observed in the *KIT* gene sequence reported in the oryCun2.0 genome version derived from an inbreed albino Thorbecke rabbit. Haplotype 2 was homozygous in all F1 *en/en* rabbits, except for one polymorphic position (g.93951861G>A) which was heterozygous in just one animal (Table S3). All polymorphisms were also observed in other rabbits of different breeds but in different haplotype combinations (data not shown). Based on these results, haplotype 1 reported in this study can be considered the *English spotting* haplotype which in heterozygosity determines the Checkered coat color pattern. When this haplotype is present in the homozygous condition, it produces a reduction in colored regions and the associated megacolon defect.

### KIT gene expression

Reverse transcription analyses of the *KIT* cDNA in both *En/En* and *en/en* rabbits confirmed that none of the variants present in the two major haplotypes (haplotype 1 and haplotype 2) affect splicing processes. Transcript sequences obtained from colon RNA for 6 overlapping cDNA regions had the same size in both *En/En* and *en/en* rabbits. In addition, direct sequence analysis of the entire transcript excluded the presence of any splice defect associated with any variants identified in *En/En* animals (EMBL/GenBank accession number KJ495954).

**Figure 4:**
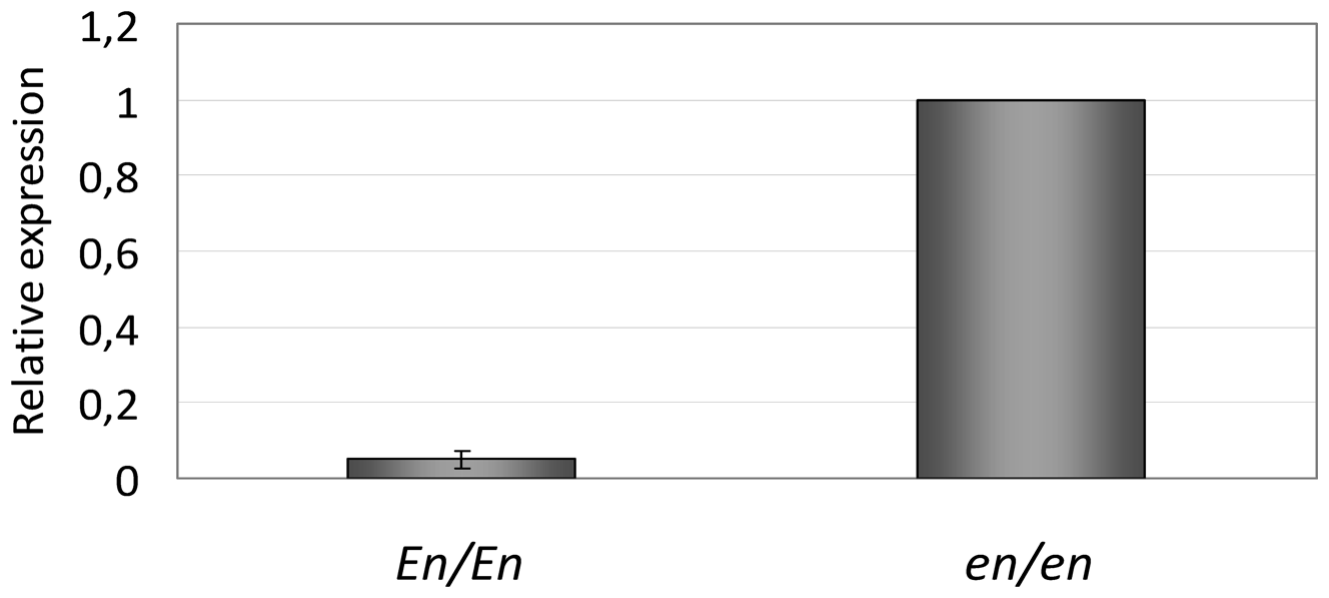
Relative *KIT* gene expression levels in the ascending colon of *En/En* and *en/en* rabbits. In *En/En* rabbits *KIT* expression was significantly lower than in *en/en* rabbits (5±2%; *P*<0.00001, *t*-test).

To test whether the level of *KIT* gene expression was altered in *En/En* animals compared to normal *en/en* rabbits, we performed qPCR on total RNA extracted from the ascending colon. *KIT* expression was markedly reduced in *En/En* rabbits. In these animals, *KIT* transcript levels for all primer pairs tested was significantly lower than in *en/en* rabbits (*P*<0.00001; Figure 4). The reduction in *KIT* expression was confirmed in the cecum of *En/En* animals where its level was reduced to 11% compared to *en/en* rabbits (data not shown).

### C-kit immunoreactive interstitial cells of Cajal (ICC)

**Figure 5:**
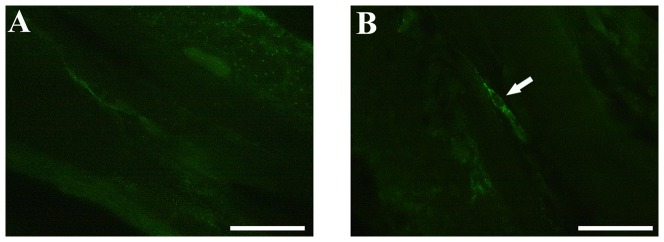
Representative images of rabbit colonic mucosa in *En/En* vs *en/en*. **A**) *En/En*; **B**) *en/en*. The arrow indicate c-kit immunoreactivity in the *tunica muscularis* of the ascending colon in an *en/en* rabbit.

Cryosections of the ascending colon of *En/En* (pathological) and *en/en* (control) rabbits were prepared to define the distribution and organization of ICC. There was no evidence of c-kit immunoreactive (-IR) cells in the ascending colon of *En/En* rabbits, while c-kit positive cells were detected in *en/en* rabbits (Figure 5). In this latter group, c-kit positive cells were localized at the interface between circular muscle and submucosa, throughout the muscle layer, and around the myenteric plexus. Notably, c-kit positive cells surrounding myenteric plexuses had a multipolar shape with three to five primary cytoplasmic processes. These ICC formed a cellular network close to the myenteric plexus and between circular and longitudinal muscle layers. Using double labelling immunohistochemistry, we showed that c-kit-IR cells were adjacent to nNOS-IR nerve fibers running throughout the muscle layers. All these features were not detected in *En/En* rabbits.

### Immunoreactive neurons

**Figure 6:**
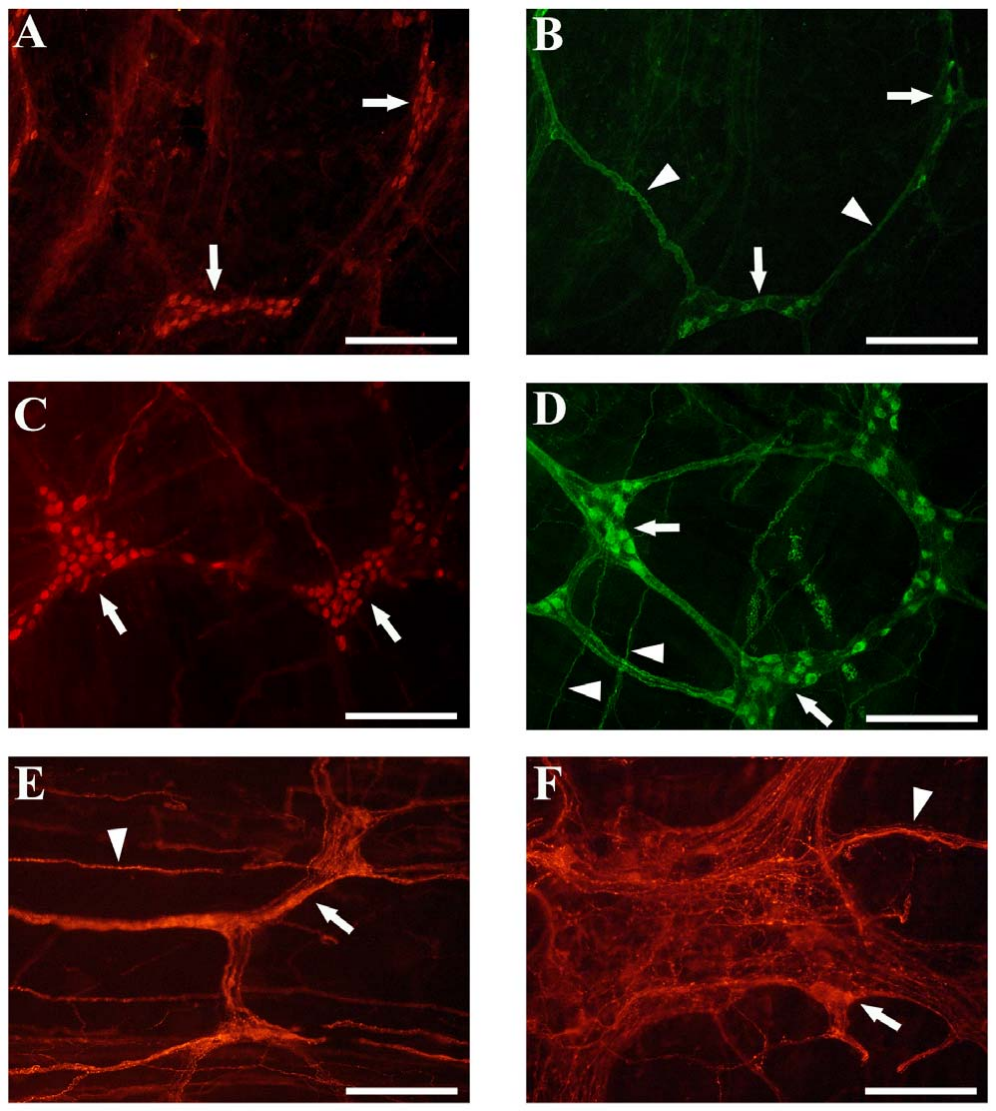
Representative images of colon whole mounts in *En/En* and *en/en* rabbit myenteric plexuses. **A**) E*n/En* myenteric ganglia (arrows) with human neuronal protein immunoreactive (HuC/D-IR) neurons. **B**) *En/En* myenteric ganglia (arrows) with nitric oxide synthase immunoreactive (nNOS-IR) neurons, (arrowheads) nNOS-IR nerve bundles. **C**) *en/en* myenteric ganglia (arrows) with HuC/D-IR neurons. **D**) e*n/en* myenteric ganglia (arrows) with nNOS-IR neurons, (arrowheads) nNOS-IR nerve bundles arranged within primary, secondary and tertiary nerve strands. In *En/En* (**A** and **B**) the ganglia and nerve bundles are less dense, have a lower number of HuC/D-IR and nNOS-IR neurons compared to *en/en* rabbits (**C** and **D**). **E**) and **F**): differences between *En/En* vs *en/en* SP-IR neurons (arrow), ganglia and nerve bundles (arrowhead) in the morphology of myenteric plexus in the ascending colon. In *En/En* (**E**) ganglia (arrow) are small and have a lower number of substance P immunoreactive (SP-IR) neurons than *en/en* rabbits (**F**). **A–D** *scale bars* 200 µm; **E–F** *scale bars* 100 µm.

**Figure 7:**
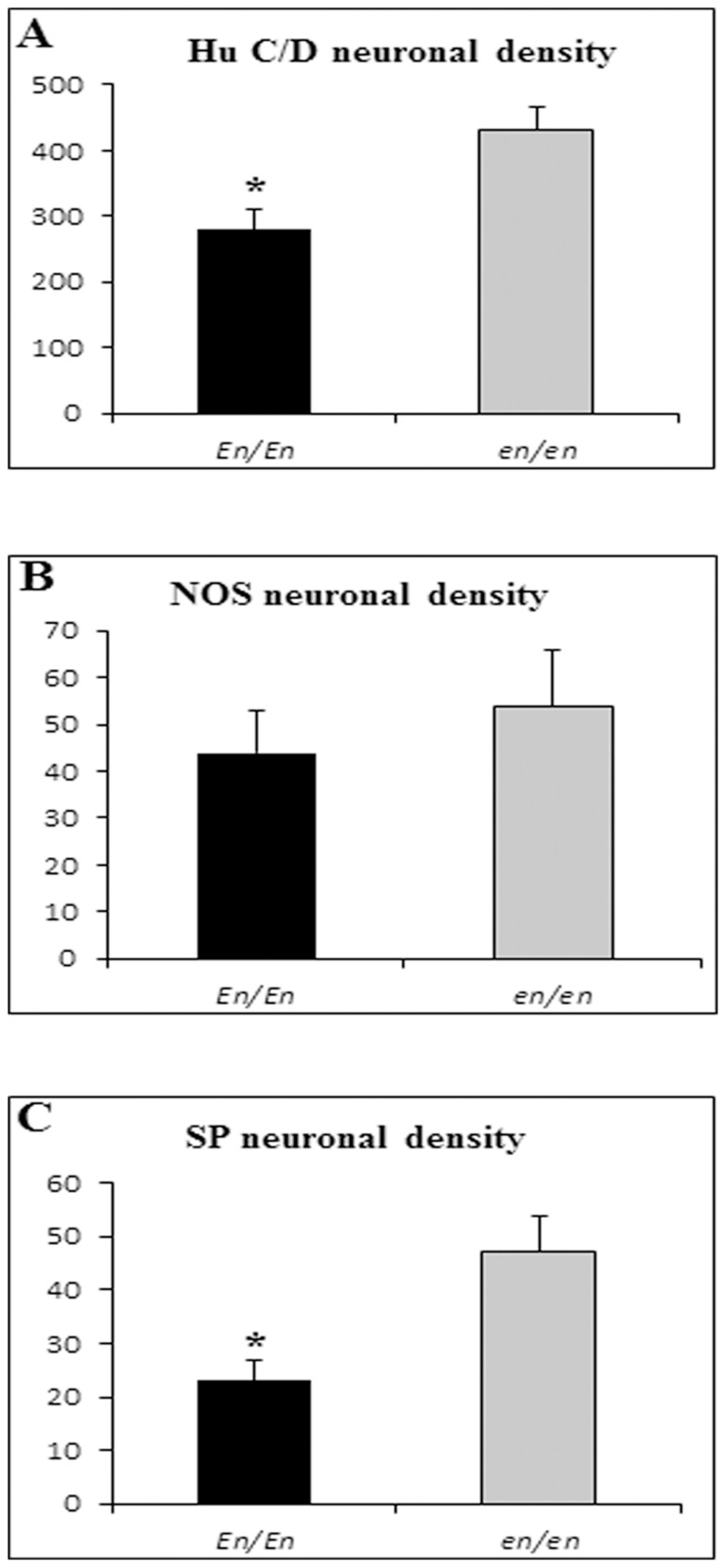
Mean density of immunoreactive myenteric neurons in *En/En* vs *en/en* rabbits. Different immunoreactive neurons are reported: human neuronal protein (HuC/D) (**A**: *En/En* vs *en/en*, *P*<0.05), nitric oxide synthase (nNOS) (**B**) and substance P (SP) (**C**: *En/En* vs *en/en*, *P*<0.01) myenteric labeled neurons. Results are expressed as mean ± standard error of the mean.

The pan-neuronal marker (HuC/D) was used to label all neuronal bodies in the myenteric plexus of the ascending colon of each rabbit. HuC/D-IR neurons were organized in ganglia of small and large sizes and showed a polygonal or elongated shape (Figures 6A, C and D). Morphometric assessment showed a statistically significant (*P*<0.05) decrease in the density of HuC/D labeled myenteric neurons in *En/En* (mean ± SEM: 280±35, n. = 4) compared to *en/en* (430±35, n. = 6) rabbits (Figure 7A).

No qualitative changes were observed between nNOS neurons of *En/En* and *en/en* rabbits. nNOS-IR was detected in the cytoplasm and the axon of myenteric neurons and, occasionally, in some short lamellar processes arising from somata of irregular profile. nNOS-IR nerve bundles were arranged within the primary, secondary and tertiary nerve strands (Figures 6B and D). nNOS-IR fibers showed varicosities along the smallest secondary and tertiary interconnecting nerve strands and within the ganglia in which they made baskets around both nNOS-negative and -positive neurons. nNOS-IR neurons were detected in virtually all myenteric plexuses. The quantitative analysis showed no difference between the density of nNOS-IR myenteric neurons in *En/En* (44±9) vs *en/en* (54±12) rabbits (Figure 7B).

There were no apparent morphological changes in SP-containing neurons between *En/En* vs. *en/en* rabbits. SP-IR neurons were observed in the ganglia of the myenteric plexus, forming a dense network of nerve bundles with small varicosities. Some SP-IR nerve fibers were seen to wrap around myenteric ganglia forming basket-like structures. Small clusters of two to four neurons or isolated neurons were seen dispersed along thin bundles (Figures 6E and F). SP-IR neurons displayed great variability both in size and shape. Morphometric data showed a statistically significant decrease in the density of SP-IR neurons in *En/En* (47±7) vs *en/en* (76±14; *P*<0.01) rabbits (Figure 7C).

Isolated CGRP-IR neurons (1 or 2 neurons) were observed in myenteric ganglia and, occasionally, dispersed along the course of nerve fibers. The density of CGRP-IR neurons was 2.4±1 and 3.8±2 in *En/En* vs *en/en* rabbits, respectively, with no significant difference due to the small number of quantifiable CGRP-IR neurons.

### Electron microscopy

**Figure 8:**
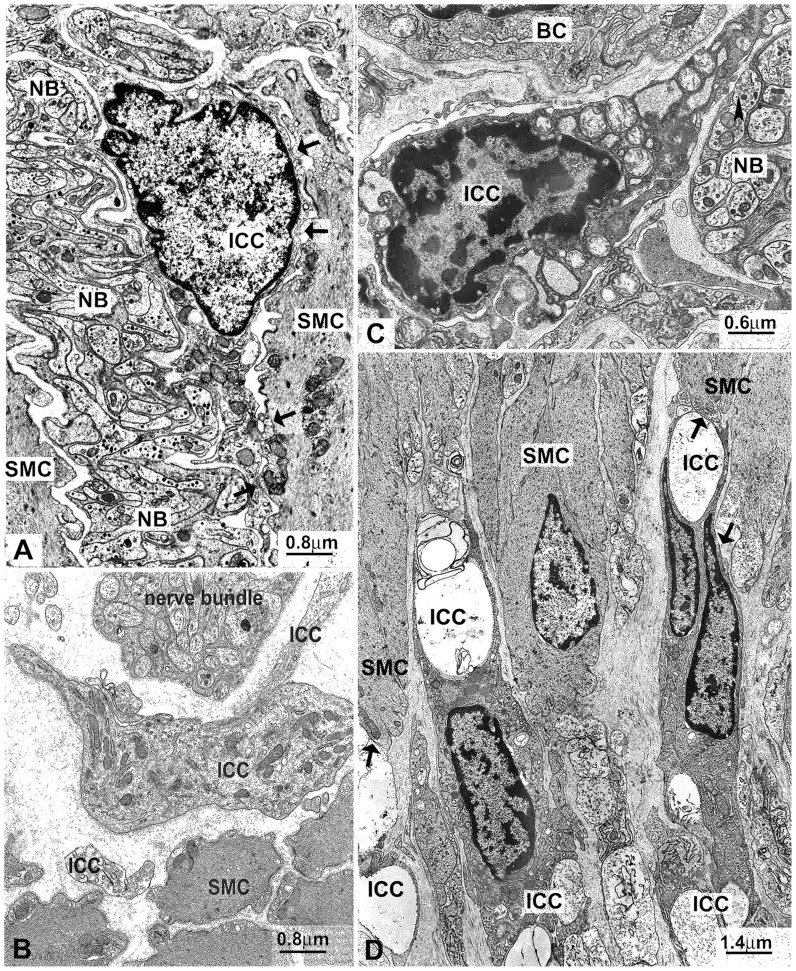
Electron microscopy of Interstitial cells of Cajal (ICC). *en/en* (**A** and **B**) control and *En/En* (**C** and **D**) pathological animals. **A**) ascending colon: an intramuscular ICC close to a nerve bundle (NB) and smooth muscle cells (SMC). Arrows indicate cell-to-cell contacts between ICC and SMC. x12,500. **B**) ascending colon: ICC at the submucosal border of the circular muscle layer showing in the cytoplasm several cisternae of the smooth endoplasmic reticulum and filaments, and caveolae along the plasma membrane; on the upper side, a nerve bundle and, on the lower side, the smooth muscle cells (SMC). x12,500. **C**) ascending colon: an intramuscular ICC with swollen mitochondria and extremely dilated cisternae of rough endoplasmic reticulum; on the upper side, a blood capillary (BC); on the right side, a nerve bundle (NB). The arrowhead indicates a nerve ending near the ICC. x15.000. **D**) descending colon: several intramuscular ICC with large intracytoplasmatic vacuoles; the arrows indicate cell-to-cell contacts between ICC and SMC. x7,500.

Electron microscopy analysis revealed that ICC and some neurons showed several significant alterations in the colon of *En/En* rabbits, but not of *en/en* rabbits. In the latter, ICC were found in the myenteric plexus area, the submucosal border of the circular muscle layer and intramuscularly. Typically, they were all near or in close contact with both smooth muscle cells and nerve endings (Figures 8A and B). The cytoplasm contained several small and elongated mitochondria, long and thin cisternae of smooth endoplasmic reticulum, few cisternae of rough endoplasmic reticulum (RER) and intermediate filaments, and several caveolae were distributed along the plasma membrane (Figures 8A and B). Conversely, in the entire (especially the ascending) colon of *En/En* rabbits (pathological), ICC were rarely found and most of them had severely altered features, including extremely dilated RER cisternae, swollen mitochondria, large vacuoles and lamellar bodies (Figures 8C and D). Caveolae were not detected. Cell-to-cell contact with smooth muscle cells was maintained (Figure 8D), while contacts between nerve endings and ICC were rarely seen (Figure 8C).

**Figure 9:**
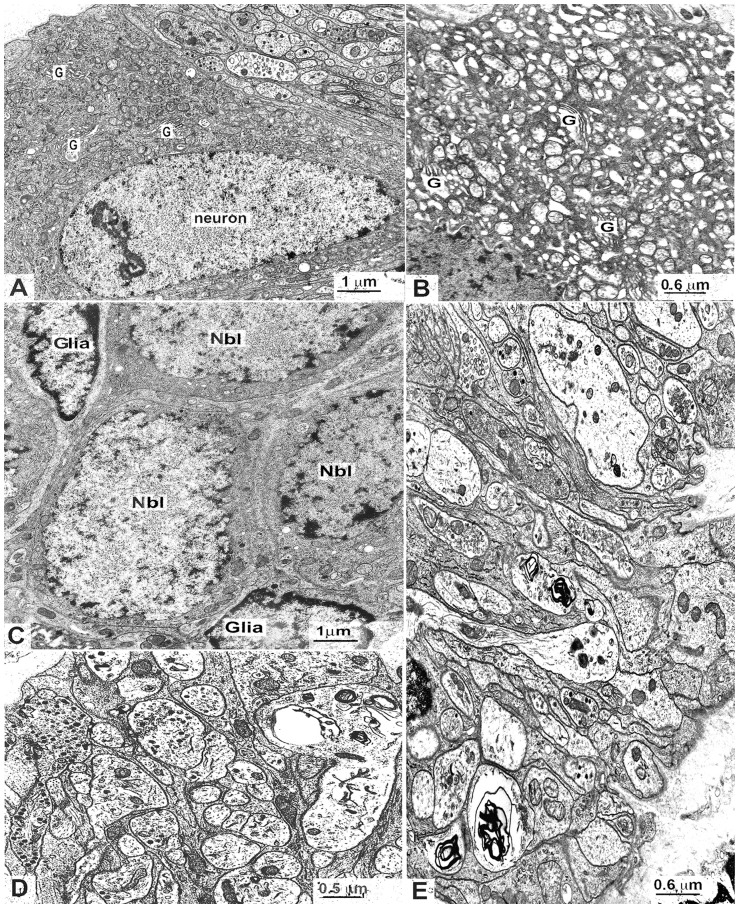
Electron microscopy of myenteric neurons. *en/en* control (**A**) and *En/En* (**B** and **C**) pathological animals. **A**) descending colon: the neuron has an extended Golgi apparatus (G), thin RER cisternae, free ribosomes, and small oval shaped mitochondria. x10,000. **B**) descending colon: detail of the cytoplasm of a neuron with dilated RER cisternae and swollen mitochondria; G: Golgi apparatus. x15,000. **C**) ascending colon: a myenteric ganglion with three cells identifiable as neuroblasts (Nbl). These cells are small and have a scarce cytoplasm mainly containing free ribosomes. Glia: glial cells. x10,000. Intragangliar nerve endings (NE): pathological animals. **D**) descending and **E**) ascending colon. Most of the NE has an anomalous content: few or abnormally featured synaptic vesicles, lamellar bodies, lysosomes. **D**) x15,000; **E**) x20,000.

In *en/en* rabbits (Figure 9A), myenteric neurons had a typically extended Golgi apparatus, small and oval-shaped mitochondria distributed throughout the perikaryon, flattened RER cisternae and free ribosomes. The cytoskeleton consisted of regularly arranged neurofilaments and microtubules. In *En/En* rabbits, some myenteric neurons that showed an abnormally extended RER with dilated cisternae and swollen mitochondria were found in close proximity to others with an apparently normal shape (Figure 9B). In the ascending colon, small cells, identifiable as neuroblasts (because of the cytoplasm filled with free ribosomes, few RER cisternae and a small Golgi apparatus), were detected (Figure 9C). In contrast with *en/en* rabbits (Figure 8A), *En/En* rabbits showed most of the nerve endings containing few synaptic vesicles, lamellar bodies, lysosomes and abnormal vesicles (Figures 9D and E).

## Discussion

As described for several livestock and animal models, selection pressures resulting from domestication and controlled breeding have combined to produce a great genetic variability in coat color [45]. This diversity is a valuable natural genetic resource to understand the biological mechanisms underlying pigmentation and associated phenotypes. In this study we analysed the *English spotting* coat color locus in Checkered Giant rabbits associated with a pathological state that could be a natural genetic model of megacolon, and showed that this phenotype is caused by mutational event(s) affecting the *KIT* gene.

Classical genetic studies in rabbits have established linkage between the *angora* (affecting hair length), the *Dutch* (determining the characteristic coat color Dutch pattern, see Fig. S1 in [7]) and the *English spotting* loci [10]. These loci belong to Linkage Group II [46], subsequently anchored to rabbit chromosome 15 (OCU15) using a microsatellite linkage map [47]. A strong candidate for the *angora* locus, the fibroblast growth factor 5 (*FGF5*) gene, already shown to be associated with hair length in other mammals [48], [49] and strongly linked to this locus in rabbit [50], is located between nucleotides 70101250–70122848 on OCU15 (ENSEMBL accession no. ENSOCUT00000012415 in the oryCun2.0 rabbit genome version). The rabbit *KIT* gene is located on the same chromosome on position 93911537–93952727, confirming, indirectly, classical linkage data showing that the *angora* and *English spotting* loci were about 20 cM apart to each other [46]. The *Dutch* and the *English spotting* loci have been described to be very close to each other or to be different allelic forms of the same locus that could have an extended range of variation with different Dutch-English spotted grades [2], [10]. It could be possible that different mutations affecting the *KIT* gene could produce different coat color patterns in rabbits as already reported for other species. For example, in pigs, a complex series of variants caused by copy number variation including and/or regulating the *KIT* gene and several other mutations are responsible for the *dominant white* locus that includes alleles determining completely white, spotted, belted and roan phenotypes [29], [31], [35], [51]. In the current version of the rabbit genome (oryCun2.0), the promoter region of the rabbit *KIT* gene is missing. We speculate that complex regulatory mutations affecting this region (and eventually other regions), may account for the large variability associated with the spotted phenotypes related to OCU15 (from *Dutch* to *English spotting* with a continuum of black and white grades [2]), including the Checkered spotted pattern. However, at present we can only deduce indirectly from our genotyping data of the g.93948587T>C SNP (Table 1) that the *En*-*Checkered* allele, the *En* allele of the English spot breed, and possibly the supposed *En*-*Dutch* allele(s) (as few Dutch rabbits have been genotyped) could derive from different *KIT* mutations. Further studies investigating the *KIT* gene in Dutch and English Spot rabbit breeds are needed to confirm this hypothesis.

Resequencing of the entire coding region, the 5′- and 3′-flanking regions and the adjacent intron boundaries of the rabbit *KIT* gene revealed the presence of 98 genetic variants. None of them have an impact on correct functionality of the encoded protein or splicing of this gene as predicted by *in silico* analysis and demonstrated by transcript analysis in *En/En*, *En/en* and *en/en* rabbits. Although we could not identify the causative variant in the rabbit *KIT* gene responsible for the spotted phenotype, expression analyses in colon and cecum support the role of this gene in determining the associated megacolon defect of the *English spotting* locus in Checkered Giant rabbits. In *En/En* animals *KIT* expression was almost completely absent compared to control *en/en* rabbits, further supporting the involvement of a regulatory variant in this locus. These *KIT* gene expression results can establish a link between this coat color locus and altered functionality of the ICC, that may be involved in determining the megacolon defect in *En/En* rabbits.

The term ‘megacolon’ is used to indicate a marked dilatation of the colon (mainly cecum and/or the sigmoid) due to a severe dysmotility affecting the large bowel. Although rare, this condition has still unclear pathogenetic aspects partly due to the few animal models available so far. Digestive motility depends on the function of different specialized cells, i.e. smooth muscle contractility and the related pacemaker activity evoked by ICCs, both finely tuned by intrinsic (the ENS) and extrinsic (sympathetic and parasympathetic) nerves [52]. Disturbances in digestive motility can occur as results of a variety of abnormalities affecting each of these elements (alone or in combination) involved in the physiology of gut motor function. From a pathologic stand point, a megacolon can be classified in aganglionic and non-aganglionic phenotypes. In the former type, the dilatation occurs above the aganglionic segment (usually extending up from the distal colon) and characterized by the lack of enteric ganglia, the histopathological hallmark of Hirschsprung disease. In the latter type, the non-aganglionic megacolon results from abnormalities (degeneration and loss, but not lack) of enteric neurons or extrinsic nerves usually secondary to a variety of conditions such as Chagas' and neurodegenerative diseases (e.g. Parkinson's and Alzheimer's diseases) [53]–[55]. Most if not all models studied so far pertain to the aganglionic megacolon, while little is known about animals characterized by a phenotype reminiscent of a non-aganglionic megacolon.

In this context, we have provided evidence of a number of neuronal and ICC changes in the ascending colon of the *En/En* rabbit model studied herein. Indeed, compared to control *en/en*, the total number of enteric neurons in the *En/En* ascending colon was found to be significantly reduced and this finding contributes to explain the altered motility and dilatation observed in this rabbit model. In particular, there was a significant decrease of the excitatory (i.e. tachykininergic) neuronal component and a trend in the decrease of the inhibitory (i.e. nitrergic) neuronal component. Taken together these results may explain the megacolon phenotype observed in this model. Furthermore, electron microscopy analysis demonstrated the presence of occasional immature rather than degenerated, myenteric neurons which suggests that a developmental, genetically-driven event may play a role in the ENS changes described in this rabbit model. Indeed, ancillary data support the neuropathological findings, since *En/En* rabbits showed hard stools in the distended ascending colon and an increased overall mortality *vs* controls (data not shown).

In addition to neuronal abnormalities, we found alterations of the ICC network in the ascending colon of the *En/En* model. Based on the consistent data showing that ICC play a prominent role in gastrointestinal (GI) motility as they generate pace-maker activity, regulate neuronal input to smooth muscle cells, and contribute to some sensory function [22], [23] we showed reduced and altered c-kit immunolabeled ICC in *En/En* rabbits *vs* controls. The ICC alteration, together with the characteristically spotted coat colors, further support a role of the *KIT* gene in the pathogenetic determination of this phenotype. The *KIT* gene is an ideal candidate for this model since, in compound *Kit* heterozygous mice (*W/W^V^* and *Sl/Sl^d^* animals) with partial loss-of-function mutations in *Kit* receptors or the *Kit* ligand, ICC fail to develop in various regions of the GI tract. Signalling via the KIT receptor is fundamental for the development and maintenance of ICC functions in the GI tract [56]. The ICC alterations in our model are reminiscent of some severe dysmotility disorders in humans. Specifically, abnormal ICC networks have been reported in tissue specimens of patients with Hirschsprung disease [57], Chagas' megacolon [58], slow transit constipation [59], [60] and chronic intestinal pseudo-obstruction and other disorders [23], [61]–[65].

## Conclusions

The rabbit has proven to be a valuable model for different human diseases ranging from atherosclerosis to cardiovascular diseases, pulmonary diseases, osteoarthritis and neurodegenerative diseases as well for immunological and ophthalmological research [66], [67].

The model described herein of *En/En* rabbits provide a useful tool for the study of human non-aganglionic megacolon. In conclusion, our study demonstrates that combined neuronal and ICC network alterations underlie this non-aganglionic model of megacolon. *KIT* mutation(s) may account for ICC abnormalities and the subsequent motor disorder in the gut. The present findings can help understand the neuro-muscular changes occurring in human non-aganglionic megacolon, though additional studies will be needed to better characterize the mutational event in *KIT* and the specific role of ENS in this restrictive condition. Also, a more detailed characterization of this rabbit model will prove useful in elucidating the pathophysiology of human megacolon.

The identification of the *KIT* gene as the causative gene in the *English spotting* locus in Checkered Giant rabbits contributes to understand the genetic basis of coat color in this species and prompts additional studies to characterize the large variability of spotted phenotypes available in several rabbit breeds. It will be very interesting to evaluate if other alleles at this locus, i.e. the *En* allele of the English spot breed, could show similar neuronal and ICC changes in GI tracts to those observed in *En/En* Checkered animals. The *English spotting* locus has the potential to establish several rabbit megacolon models based on different natural mutations of the *KIT* gene.

## Supporting Information

Figure S1 **Schematic representation of the rabbit** ***KIT*** **gene with sequenced regions.** Boxed regions  =  exons reported in the oryCun2.0 gene sequence (Ensembl accession number ENSOCUG00000007086). Sequenced regions are indicated with lines below the gene structure. The position of the genotyped polymorphism (g.93948587T>C) is reported. To produce a complete representation of the gene, intronic and exonic (boxed) regions are not on scale.
(TIF)

Figure S2 **Viscera of an** ***En/En*** **rabbit (70 days old) showing clear signs of megacolon with hard feces.**
(TIF)

Table S1 **PCR primers, PCR conditions and use of the obtained PCR primers and products.**
(DOC)

Table S2 **List of antibodies used in this study and their respective working dilutions.**
(DOC)

Table S3 **Polymorphisms identified in the rabbit** ***KIT*** **gene.**
(DOC)
