# Supplementary figures and images for: The KIT Gene Is Associated with the English Spotting Coat Color Locus and Congenital Megacolon in Checkered Giant Rabbits (Oryctolagus cuniculus)
Source: PLoS One. 2014 Apr 15;9(4):e93750. doi: 10.1371/journal.pone.0093750 (PMC3988019; doi:10.1371/journal.pone.0093750)

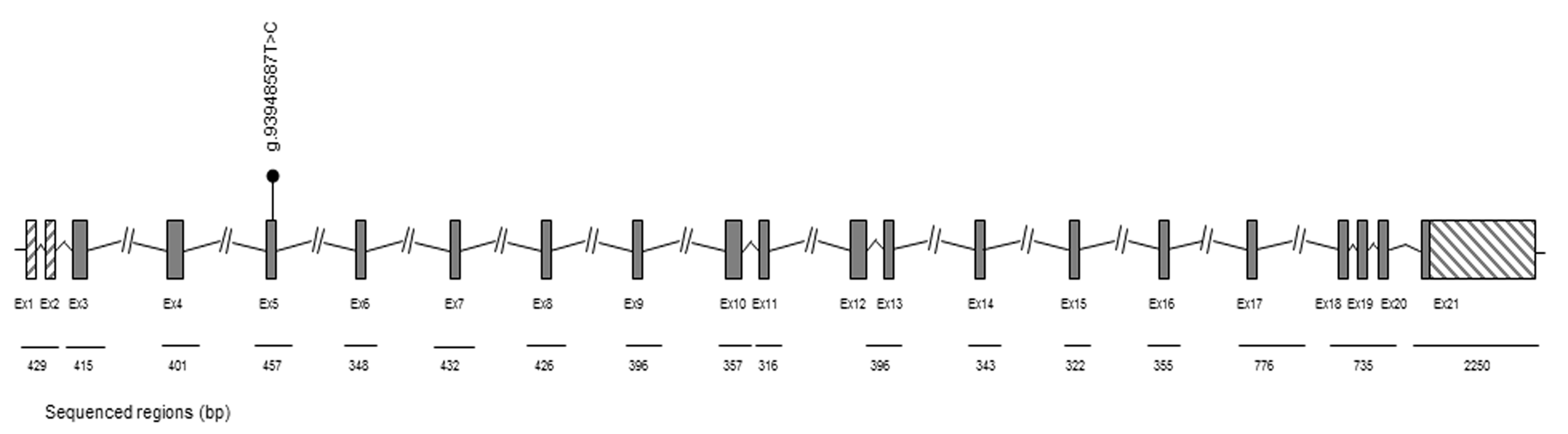

Supplement: Figure S1 — Schematic representation of the rabbit KIT gene with sequenced regions. Boxed regions = exons reported in the oryCun2.0 gene sequence (Ensembl accession number ENSOCUG00000007086). Sequenced regions are indicated with lines below the gene structure. The position of the genotyped polymorphism (g.93948587T>C) is reported. To produce a complete representation of the gene, intronic and exonic (boxed) regions are not on scale. (TIF) [file pone.0093750.s001.tif]

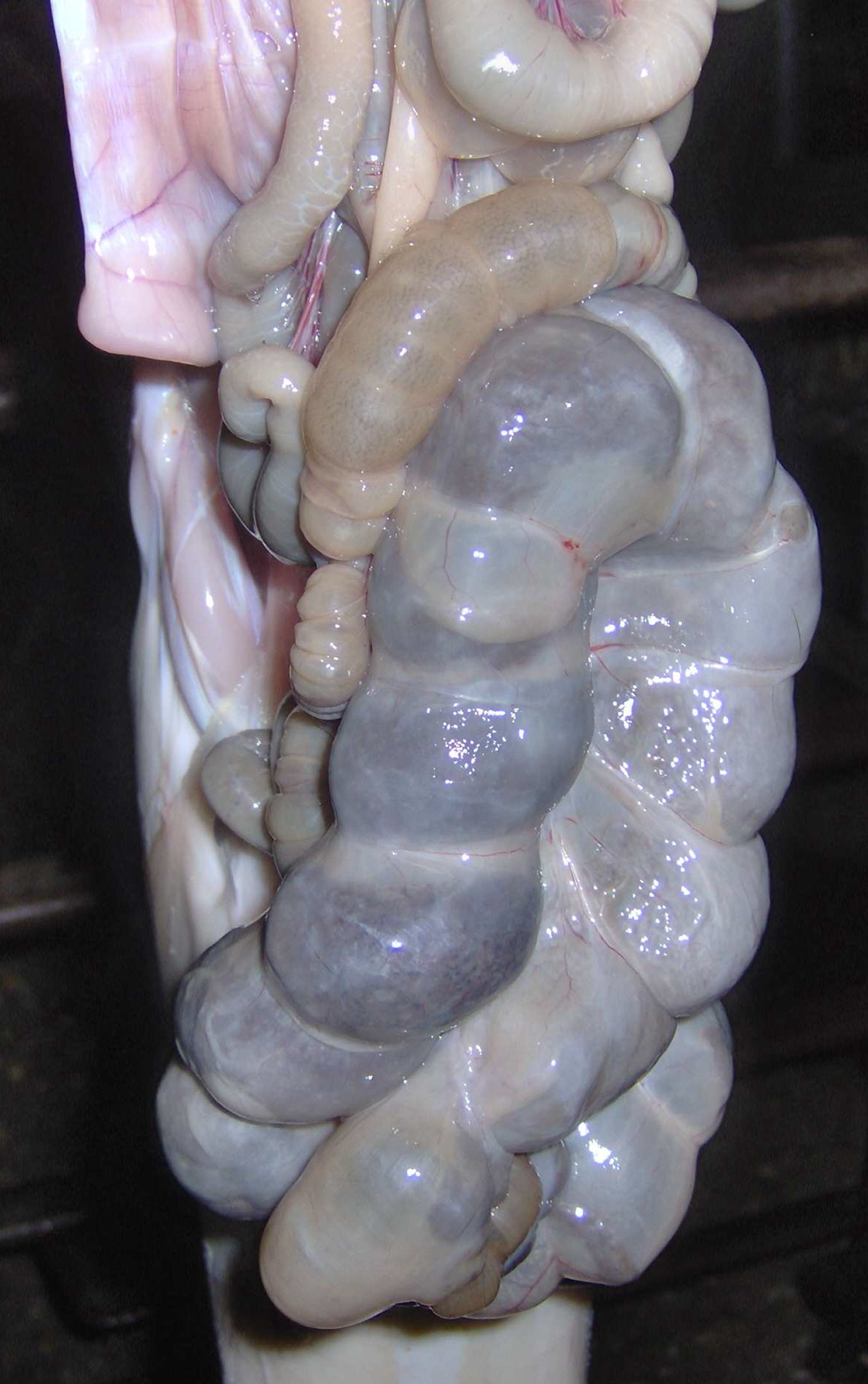

Supplement: Figure S2 — Viscera of an En/En rabbit (70 days old) showing clear signs of megacolon with hard feces. (TIF) [file pone.0093750.s002.tif]
