# Supplementary material for: The KIT Gene Is Associated with the English Spotting Coat Color Locus and Congenital Megacolon in Checkered Giant Rabbits (Oryctolagus cuniculus)
Source: PLoS One. 2014 Apr 15;9(4):e93750. doi: 10.1371/journal.pone.0093750 (PMC3988019; doi:10.1371/journal.pone.0093750)
Supplement: Table S1 — PCR primers, PCR conditions and use of the obtained PCR primers and products. (DOC) [file pone.0093750.s003.doc]

**Table S1.** PCR primers, PCR conditions and use of the obtained PCR primers and products.

| **Primer pair name** | **Primer forward (5’-3’)** | **Primer reverse (5’-3’)** | **Gene region or reference/size^1^** | **PCR conditions^2^** | **Use^3^** |
| --- | --- | --- | --- | --- | --- |
| KIT_1-2 | CTTCGTCCCACTGCTCCTG | CACGAGGGAGAAGAAACAGC | Part of the 5'-UTR/flanking region, exon 1, intron 1, exon 2 and part of intron 2/468 bp | 58/2.5/EuroTaq | Sequencing |
| KIT_3 | GCCCTAAATAGCAGGACAGC | GCATCTGCGTGTCAAGATCA | Part of intron 2, exon 3, and part of intron 3/455 bp | 56/2.0/EuroTaq | Sequencing |
| KIT_4 | ACAGCCATGCGAACCACT | CTCTCCAACAGAGGCTCCAC | Part of intron 3, exon 4, and part of intron 4/439 bp | 62/3.0/EuroTaq | Sequencing |
| KIT_5 | TTAGTGCCGGATTCCAAGAC | CCTTCGATACGGAAGGATCA | Part of intron 4, exon 5, and part of intron 5/497 bp | 58/2.5/EuroTaq | Sequencing, PCR-RFLP (*Fsp*BI) |
| KIT_6 | TGAATATTAATGGTATGGTAATTCTGG | TGCAGTGATATTTTCATTCATTCAT | Part of intron 5, exon 6, and part of intron 6/400 bp | 56/2.5/AmpliTaq Gold | Sequencing |
| KIT_7 | CGGTGATTTTATAAGAACGGAAG | CAGAGCCGTGGAGTGAGAG | Part of intron 6, exon 7, and part of intron 7/474 bp | 59/3.5/EuroTaq | Sequencing |
| KIT_8 | GGCGTGTGTTTGCTTAGGAG | TGCTTTCCCATTCTCTGGAC | Part of intron 7, exon 8, and part of intron 8/466 bp | 57/2.5/EuroTaq | Sequencing |
| KIT_9 | AGGTTTCCAGCAGTCTGACG | CGAATTACCCTGTCCTGGAA | Part of intron 8, exon 9, and part of intron 9/436 bp | 59/1.5/EuroTaq | Sequencing |
| KIT_10 | GAGCGGGAGTCGGATTGT | GGTGGGATGGGTGTTATCAA | Part of intron 9, exon 10, and part of intron 10/395 bp | 61/2.5/EuroTaq | Sequencing |
| KIT_11 | CATCGTCATCCATCTGTCGT | GGAGGAAGCTTCTGGCTCTT | Part of intron 10, exon 11, and part of intron 11/356 bp | 58/2.5/EuroTaq | Sequencing |
| KIT_12-13 | CCCTGGGCGCCGGCGCCTT | GCCCTGGGTCCTGGGCTACG | Part of exon 12, intron 12, exon 13 and part of intron 13/435 bp | 59/2.5/EuroTaq | Sequencing |
| KIT_14 | CTATGATTGTGCAGGGACCA | GGGTGCCTCAGTGTGTTTT | Part of intron 13, exon 14, and part of intron 14/382 bp | 54/1.5/AmpliTaq Gold | Sequencing |
| KIT_15 | TTCTTTGTGTGTCGCTCGTC | GAGCCACCACCACCGTCT | Part of intron 14, exon 15, and part of intron 15/360 bp | 58/2.5/EuroTaq | Sequencing |
| KIT_16 | CCGTCTTCACAGGCCCTAC | GCTCATTTAGGCAGTTTGCAC | Part of intron 15, exon 16, and part of intron 16/392 bp | 56/2.5/AmpliTaq Gold | Sequencing |
| KIT_17 | GCTAGTCCCGAGAACACGAG | AGAATTCGGGACTCTGACCA | Part of intron 16, exon 17, and part of intron 17/467 bp | 58/2.5/AmpliTaq Gold | Sequencing |
| KIT_17-b | CTCCAGGCCAACTTGCAT | CGGCTGCCTTAGGATTCTC | Part of intron 17/385 bp | 58/2.5/EuroTaq | Sequencing |
| KIT_18-19 | ATTGTGAGCTCGGCAGTC | ACGGAGAGACGGCAAAGG | Part of intron 17, exon 18, intron 18, exon 19, and part of intron 19/394 bp | 58/1.5/AmpliTaq Gold | Sequencing |
| KIT_19-20 | CCGGTGGACTCCAAGTTCTA | AGGAAACACGGTGAAC | Part of exon 19,intron 20, exon 20 and part of intron 20/480 bp | 56/2.5/AmpliTaq Gold | Sequencing |
| KIT_21-1 | ACAGGGACCCTCGCTGAC | GAGGACAGAATCGGCAAGTC | Part of intron 20, exon 21 (CDS), part of 3'-UTR/426 bp | 58/3.0/EuroTaq | Sequencing |
| KIT_21-2 | CAGGCCACAGAAAAAGTGTG | GTGGTGACAGAAGCCAGACC | Part of 3'-UTR/395 bp | 58/2.5/EuroTaq | Sequencing |
| KIT_21-3 | GCTCAGCTGACCTAGCGTGT | AGGACACCACCCCCACTATT | Part of 3'-UTR/470 bp | 61/2.5/EuroTaq | Sequencing |
| KIT_21-4 | TGGGTTTAGCCGAATAGTGG | TTCAGACACATCTGGCCAAC | Part of 3'-UTR/492 bp | 57/2.5/EuroTaq | Sequencing |
| KIT_21-5 | ACTCGTGGGCTTGGGAATA | GTGGCCAGGTTTAGGATTGG | Part of 3'-UTR/474 bp | 64/2.5/EuroTaq | Sequencing |
| KIT_21-6 | TTGAAATGTAGCAATAATGTCTTTTG | GCACAGGAAGCTGAGTCTGA | Part of 3'-UTR/472 bp | 60/2.5/EuroTaq | Sequencing |
| KIT_cDNA_1 | AGCAGGTTCTCGCTGGAGT | AGGCAGCTCGTCCTAGACAC | Part of exon 3, exon 4 and part of exon 5/512/bp | 60/GoTaq | RT-PCR, Sequencing |
| KIT_cDNA_2 | CCAAGGACTTGACCTTCGTC | CCACCAAGTCCACGTTTTCT | Part of exon 4, exons 5 and 6, part of exon 7/527 bp | 60/GoTaq | RT-PCR, qPCR, Sequencing |
| KIT_cDNA_3 | ATCAGGGCGACTTCCACTAC | GAATCCTGCTGCCTCACACT | Part of exon 6, exons 7 and 8, part of exon 9/512 bp | 60/GoTaq | RT-PCR, Sequencing |
| KIT_cDNA_4 | CGTGAACACGAAACCAAAGA | AGTTCAGGAGGTCCCCGTAG | Part of exon 9, exons 10, 11, 12, 13, and part of exon 14/791 bp | 60/GoTaq | RT-PCR, Sequencing |
| KIT_cDNA_5 | CTACGGGGACCTCCTGAACT | CGTGGGTAAGGAGGATGTTC | Part of exon 14, exons 15 and 16, part of exon 17/386 bp | 60/GoTaq | RT-PCR, qPCR, Sequencing |
| KIT_cDNA_6 | GGACCTGGAGGACTTGTTGA | TCTCTGAGATCTGCGTCTCG | Part of exon 16, exons 17, 18, and 19, and part of exon 20/500 bp | 60/GoTaq | RT-PCR, qPCR, Sequencing |
| HPRT | TGATAGATCCATTCCTATGACTGTAGA | GGGTCCTTTTCACCAGCAG | Godornes et al. (2007)/256 bp | 60/GoTaq | qPCR |

^1^ Amplified gene regions and amplified fragment size.

^2^ Annealing temperature °C / MgCl_2_ concentration (where applicable)/*Taq* DNA polymerase

^3^ Use of the amplified fragments or primer pairs.
