# Supplementary material for: The KIT Gene Is Associated with the English Spotting Coat Color Locus and Congenital Megacolon in Checkered Giant Rabbits (Oryctolagus cuniculus)
Source: PLoS One. 2014 Apr 15;9(4):e93750. doi: 10.1371/journal.pone.0093750 (PMC3988019; doi:10.1371/journal.pone.0093750)
Supplement: Table S2 — List of antibodies used in this study and their respective working dilutions. (DOC) [file pone.0093750.s004.doc]

**Table S2. List of antibodies used in this study and their respective working dilutions.**

| **Type of antisera** | **Antisera** | **Species** | **Code** | **Dilution** | **Supplier** |
| --- | --- | --- | --- | --- | --- |
| Primary antisera | Human neuronal protein (HuC/D) | Goat | sc-5977 | 1:50 | Santa Cruz |
|  | Neuronal nitric oxide synthase (nNOS) | Mouse | sc-5302 | 1:200 | Santa Cruz |
|  | Substance P (SP) | Rat | 10-S15A | 1:50 | Fitzgerald |
|  | Calcitonin gene-related peptide (CGRP) | Mouse | sc-57053 | 1:150 | Santa Cruz |
|  | c-Kit | Goat | sc-1493 | 1:100 | Santa Cruz |
| Secondary antisera | FITC-conjugated donkey anti-mouse IgG | - | - | 1:200 | Jackson |
|  | TRITC-conjugated donkey anti-goat IgG | - | - | 1:300 | Jackson |
|  | Alexa 488-conjugated donkey anti-goat IgG | - | - | 1:400 | Molecular Probes |
|  | Alexa 594-conjugated donkey anti-rat IgG | - | - | 1:400 | Molecular Probes |
