# Supplementary material for: The KIT Gene Is Associated with the English Spotting Coat Color Locus and Congenital Megacolon in Checkered Giant Rabbits (Oryctolagus cuniculus)
Source: PLoS One. 2014 Apr 15;9(4):e93750. doi: 10.1371/journal.pone.0093750 (PMC3988019; doi:10.1371/journal.pone.0093750)
Supplement: Table S3 — Polymorphisms identified in the rabbit KIT gene. (DOC) [file pone.0093750.s005.doc]

**Table S3.** Polymorphisms identified in the rabbit *KIT* gene.

| **Polymorphisms^1^** | **Type of polymorphism^2^** | **Gene region^3^** | **Haplotype in oryCun2.0** | **Haplotype 1 (*En*)** | **Haplotype 2 (*en*)** | **Aminoacid substitution^4^** |
| --- | --- | --- | --- | --- | --- | --- |
| g.93952714G>A | SNP | Exon 1 | G | G | G | Non coding region |
| g.93952650G>C | SNP | Intron 1 | G | G | C | - |
| g.93952644delT | indel | Intron 1 | T | T | delT | - |
| g.93952625A>G | SNP | Intron 1 | A | A | A/G | - |
| g.93951861G>A | SNP | Exon 3 | G | G | G/A | Synonymous |
| g.93949917G>A | SNP | Exon 4 | G | G | A | p.N213D (subPSEC = -1.569; P_deleterious_ = 0.193) |
| g.93949912C>T | SNP | Exon 4 | C | C | T | Synonymous |
| g.93949822T>C | SNP | Intron 4 | T | T | C | - |
| g.93949819C>G | SNP | Intron 4 | C | C | G | - |
| g.93948742C>T | SNP | Intron 4 | C | C | T | - |
| g.93948754T>C | SNP | Intron 4 | T | T | C | - |
| g.93948625G>A | SNP | Intron 4 | G | G | A | - |
| g.93948587T>C | SNP | Exon 5 | T | T | C | Synonymous ^5^ PCR-RFLP (*Fsp*BI) |
| g.93948584G>A | SNP | Exon 5 | G | G | A | Synonymous |
| g.93948548A>G | SNP | Exon 5 | A | A | G | Synonymous |
| g.93948400T>C | SNP | Intron 5 | T | T | C | - |
| g.93948396A>G ^6^ | SNP | Intron 5 | A | A | A | - |
| g.93948391G>C | SNP | Intron 5 | G | G | C | - |
| g.93948305G>T ^6^ | SNP | Intron 5 | G | G | G | - |
| g.93945258G>A^6^ | SNP | Exon 6 | G | G | G | Synonymous |
| g.93941902A>G ^6^ | SNP | Intron 6 | A | A | A | - |
| g.93941806G>A ^6^ | SNP | Exon 7 | G | G | G | Synonymous |
| g.93941679G>A ^6^ | SNP | Intron 7 | G | G | G | - |
| g.93941675G>A ^6^ | SNP | Intron 7 | G | G | G | - |
| g.93941626G>A ^6^ | SNP | Intron 7 | G | G | G | - |
| g.93926864A>G ^6^ | SNP | Exon 9 | A | A | A | Synonymous |
| g.93926828A>G ^6^ | SNP | Exon 9 | A | A | A | Synonymous |
| g.93926740C>G ^6^ | SNP | Intron 9 | C | C | C | - |
| g.93926671A>G ^6^ | SNP | Intron 9 | A | A | A | - |
| g.93926521C>T ^6^ | SNP | Intron 9 | C | C | C | - |
| g.93925459G>C | SNP | Intron 10 | G | G | C | - |
| g.93920495G>A | SNP | Intron 14 | G | G | G | - |
| g.93920444T>C | SNP | Intron 14 | T | T | C | - |
| g.93920382T>C | SNP | Exon 15 | T | T | C | Synonymous |
| g.93920376C>T ^6^ | SNP | Exon 15 | C | C | C | Synonymous |
| g.93920367C>T ^6^ | SNP | Exon 15 | C | C | C | Synonymous |
| g.93920291C>T | SNP | Intron 15 | C | C | T | - |
| g.93919203G>C | SNP | Intron 15 | G | G | C | - |
| g.93918962C>G | SNP | Intron 16 | C | C | G | - |
| g.93917933T>C | SNP | Exon 17 | T | T | C | Synonymous |
| g.93917761C>T | SNP | Intron 17 | C | C | T | - |
| g.93917739A>G | SNP | Intron 17 | A | A | G | - |
| g.93917675T>C | SNP | Intron 17 | T | T | C | - |
| g.93917612insGTT ^6^ | Indel GTT | Intron 17 | - | - | - | - |
| g.93917560T>C | SNP | Intron 17 | T | T | C | - |
| g.93917561T>C | SNP | Intron 17 | T | T | C | - |
| g.93917520G>A | SNP | Intron 17 | G | G | A | - |
| g.93917498G>C | SNP | Intron 17 | G | G | C | - |
| g.93917435C>T | SNP | Intron 17 | C | C | T | - |
| g.93915055A>G | SNP | Intron 18 | A | A | G | - |
| g.93915046G>A | SNP | Intron 18 | G | G | A | - |
| g.93915033G>T | SNP | Intron 18 | G | G | T | - |
| g.93915021T>C | SNP | Intron 18 | T | T | C | - |
| g.93915016T>C ^6^ | SNP | Intron 18 | T | T | T | - |
| g.93915015delACTCC | Indel | Intron 18 | ACTCC | ACTCC | - | - |
| g.93915004G>A | SNP | Intron 18 | G | G | A | - |
| g.93914997C>T ^6^ | SNP | Intron 18 | C | C | C | - |
| g.93914998G>A | SNP | Intron 18 | G | G | A | - |
| g.93914891C>T | SNP | Exon 19 | C | C | T | Synonymous |
| g.93914885T>C | SNP | Exon 19 | T | T | C | Synonymous |
| g.93914843T>C | SNP | Intron 19 | T | T | C | - |
| g.93914788G>C | SNP | Intron 19 | G | G | C | - |
| g.93914589G>C | SNP | Intron 20 | G | G | A | - |
| g.93914586C>G | SNP | Intron 20 | C | C | G | - |
| g.93914565G>A | SNP | Intron 20 | G | G | A | - |
| g.93914548G>A | SNP | Intron 20 | G | G | A | - |
| g.93913764G>A | SNP | Exon 21 | G | G | A | Synonymous |
| g.93913692_g.93913691insG | Indel | Exon 21 (3’-UTR) | - | - | insG | - |
| g.93913620A>G | SNP | Exon 21 (3’-UTR) | A | A | G | - |
| g.93913338A>G | SNP | Exon 21 (3’-UTR) | A | A | G | - |
| g.93913321A>G ^6^ | SNP | Exon 21 (3’-UTR) | A | A | A | - |
| g.93913318_93913315delAA ^6^ | Indel | Exon 21 (3’-UTR) | AA | AA | AA | - |
| g.93913309C>T | SNP | Exon 21 (3’-UTR) | C | C | T | - |
| g.93913257A>G | SNP | Exon 21 (3’-UTR) | A | A | G | - |
| g.93913255A>G ^6^ | SNP | Exon 21 (3’-UTR) | A | A | A | - |
| g.93913252G>A ^6^ | SNP | Exon 21 (3’-UTR) | G | G | G | - |
| g.93913205G>A | SNP | Exon 21 (3’-UTR) | G | G | A | - |
| g.93913201T>C | SNP | Exon 21 (3’-UTR) | T | T | C | - |
| g.93913181G>T ^6^ | SNP | Exon 21 (3’-UTR) | G | G | G | - |
| g.93913174C>T | SNP | Exon 21 (3’-UTR) | C | C | T | - |
| g.93913163G>A | SNP | Exon 21 (3’-UTR) | G | G | A | - |
| g.93913135A>G | SNP | Exon 21 (3’-UTR) | A | A | G | - |
| g.93912995G>A | SNP | Exon 21 (3’-UTR) | G | G | A | - |
| g.93912987C>T | SNP | Exon 21 (3’-UTR) | C | C | T | - |
| g.93912954C>G | SNP | Exon 21 (3’-UTR) | C | C | G | - |
| g.93912950T>C | SNP | Exon 21 (3’-UTR) | T | T | C | - |
| g.93912871G>A | SNP | Exon 21 (3’-UTR) | G | G | A | - |
| g.93912786_93912767 | Microsatellite | Exon 21 (3’-UTR) | 10 TG repeats | 10 TG repeats | 12 TG repeats | - |
| g.93912764_93912763insTA | Indel | Exon 21 (3’-UTR) | -- | -- | TA | - |
| g.93912718A>G | SNP | Exon 21 (3’-UTR) | A | A | G | - |
| g.93912640A>C | SNP | Exon 21 (3’-UTR) | A | A | C | - |
| g.93912470T>C | SNP | Exon 21 (3’-UTR) | T | T | C | - |
| g.93912385G>A | SNP | Exon 21 (3’-UTR) | G | G | A | - |
| g.93912353C>T | SNP | Exon 21 (3’-UTR) | C | C | T | - |
| g.93911840_93911832delAAAAT | Indel | Exon 21 (3’-UTR) | AAAAT | AAAAT | del | - |
| g.93911776A>G | SNP | Exon 21 (3’-UTR) | A | A | G | - |
| g.93911811A>G | SNP | Exon 21 (3’-UTR) | A | A | G | - |
| g.93911693A>C | SNP | Exon 21 (3’-UTR) | A | A | C | - |

^1^ Positions are relative to coordinate systems in Ensembl for chromosome 15 in oryCun2.0 genome version.

^2^ Single nucleotide polymorphism or insertion/deletion.

^3^ Gene regions are relative to the rabbit *KIT* gene (Ensembl number ENSOCUG00000007086) in oryCun2.0 genome version (December 2013). No information was available to clearly define 5’-UTR and 3’-UTR regions.

^4^ PANTHER results are given in parenthesis (subPSEC; P_deleterious_). PANTHER estimates the likelihood of a particular non-synonymous (amino-acid changing) coding SNP to cause a functional impact on the protein. It calculates the substitution position-specific evolutionary conservation (subPSEC) score based on an alignment of evolutionarily related proteins. The probability that a given variant will cause a deleterious effect on protein function is estimated by P_deleterious_, such that a subPSEC score of -3 corresponds to a P_deleterious_ of 0.5. The subPSEC score is the negative logarithm of the probability ratio of the wild-type and mutant amino acids at a particular position. PANTHER subPSEC scores are continuous values from 0 (neutral) to about -10 (most likely to be deleterious).

^5^ Genotyped SNP.

^6^ Polymorphisms identified by sequencing *en/en* rabbits. Polymorphisms not included in haplotype 1 or haplotype 2.
